# Modulation of subventricular zone oligodendrogenesis: a role for hemopressin?

**DOI:** 10.3389/fncel.2014.00059

**Published:** 2014-02-27

**Authors:** Sara Xapelli, Fabienne Agasse, Sofia Grade, Liliana Bernardino, Filipa F. Ribeiro, Clarissa S. Schitine, Andrea S. Heimann, Emer S. Ferro, Ana M. Sebastião, Ricardo A. De Melo Reis, João O. Malva

**Affiliations:** ^1^Center for Neuroscience and Cell Biology of Coimbra, University of CoimbraCoimbra, Portugal; ^2^Institute of Pharmacology and Neurosciences, Faculty of Medicine, University of LisbonLisboa, Portugal; ^3^Unit of Neurosciences, Institute of Molecular Medicine, University of LisbonLisboa, Portugal; ^4^Institute for Stem Cell Research, Helmholtz Centre Munich, German Research Centre for Environmental HealthNeuherberg, Germany; ^5^Department of Physiological Genomics, Faculty of Medicine, Ludwig-Maximilians University of MunichMunich, Germany; ^6^Health Sciences Research Center, University of Beira InteriorCovilhã, Portugal; ^7^Neurochemistry Laboratory, Biophysics Institute Carlos Chagas Filho, Federal University of Rio de JaneiroRio de Janeiro, Brazil; ^8^Proteimax Biotecnologia LTDA, São PauloSP, Brazil; ^9^Departamento de Biologia Celular e do Desenvolvimento, Instituto de Ciências BiomédicasSão Paulo, Brazil; ^10^Center of Investigation in Environment, Genetics and Oncobiology, Institute for Biomedical Imaging and Life Sciences, Faculty of Medicine, University of CoimbraCoimbra, Portugal

**Keywords:** oligodendrogenesis, subventricular zone, hemopressin, endocannabinoids, myelin pathologies

## Abstract

Neural stem cells (NSCs) from the subventricular zone (SVZ) have been indicated as a source of new oligodendrocytes to use in regenerative medicine for myelin pathologies. Indeed, NSCs are multipotent cells that can self-renew and differentiate into all neural cell types of the central nervous system. In normal conditions, SVZ cells are poorly oligodendrogenic, nevertheless their oligodendrogenic potential is boosted following demyelination. Importantly, progressive restriction into the oligodendrocyte fate is specified by extrinsic and intrinsic factors, endocannabinoids being one of these factors. Although a role for endocannabinoids in oligodendrogenesis has already been foreseen, selective agonists and antagonists of cannabinoids receptors produce severe adverse side effects. Herein, we show that hemopressin (Hp), a modulator of CB1 receptors, increased oligodendroglial differentiation in SVZ neural stem/progenitor cell cultures derived from neonatal mice. The original results presented in this work suggest that Hp and derivates may be of potential interest for the development of future strategies to treat demyelinating diseases.

## OLIGODENDROGENESIS

Oligodendrocytes are the myelin-forming cells of the central nervous system (CNS) of vertebrates. These glial cells possess cholesterol-rich membranes that compactly enwrap around neuronal axons building the so-called myelin sheath. This multilamellar spiral structure provides an electrical insulation and clustered distribution of ion channels in nodes and paranodes, which allows a fast and saltatory propagation of action potentials along the axon, an essential requirement for normal brain function. In addition, the myelin sheath sustains and protects the axons (reviewed in Nave and Trapp, 2008) and oligodendrocytes seem to play a role in signaling crosstalk with neurons (Bergles et al., 2010).

Oligodendrogenesis actively takes place during CNS development and following demyelinating insults, but also more silently throughout adulthood. Oligodendrocytes differentiate from oligodendrocyte precursor cells (OPCs), characterized by the presence of the neuron-glial antigen 2 (NG2) proteoglycan and therefore often called NG2 cells (Nishiyama et al., 2009). Forebrain OPCs are produced in three sequential waves during development, from different regions of the ventricular zone (VZ): at early embryonic stage (E12.5), from progenitors in the medial ganglionic eminence (MGE) and the anterior entopeduncular area (AEP); at E15.5, from progenitors in the lateral and/or caudal ganglionic eminence (LGE/CGE); and at birth (P0) from progenitors within the postnatal cortex (Kessaris et al., 2006). The highly proliferative progenitors migrate from these birth areas and colonize the developing gray and white matter. Herein, most of them exit the cell cycle and mature into myelinating oligodendrocytes. Some, however, remain as slowly dividing OPCs, widespread throughout the brain parenchyma, and constitute a stable population that comprises 3–8% of the total number of cells in the adult brain (Polito and Reynolds, 2005). Genetic fate mapping analysis has demonstrated that the progeny of these OPCs is restricted to the oligodendrocyte lineage, including new myelinating oligodendrocytes and further NG2 cells (Dimou et al., 2008; Kang et al., 2010; Clarke et al., 2012). In addition, the subventricular zone (SVZ) bordering the lateral ventricles, constitutes a source of few oligodendrocyte progenitors that migrate and populate the corpus callosum, striatum and fimbria-fornix in the adult mouse brain (Menn et al., 2006; Ortega et al., 2013).

Oligodendrogenesis in the adult brain is stimulated by myelin pathologies. Evidence indicates that most of the remyelinating oligodendrocytes are derived from the widespread parenchymal OPCs. Upon a demyelinating injury, these cells are induced to proliferate and migrate to the demyelinated area, mature and reinvest the denuded axons, forming new myelin sheaths (reviewed in Franklin and ffrench-Constant, 2008). In addition, precursors in the rodent SVZ niche become activated and contribute to remyelination of nearby callosal axonal tracts (Nait-Oumesmar et al., 1999; Picard-Riera et al., 2002; Menn et al., 2006). Noteworthy, an increased activation of the SVZ niche was detected in *post-mortem* samples from multiple sclerosis (MS) patients suggesting that a similar response may occur in the human brain (Nait-Oumesmar et al., 2007).

Although robust remyelination upon acute MS lesions results in functional recovery, as disease progresses, this spontaneous reparative process starts to fail leading to axonal degeneration and neurological deficits. In many lesions, remyelination remains restricted to the edge of the lesion (reviewed in Kotter et al., 2011). In fact, remyelination in humans varies from patient to patient or even from lesion to lesion in the same patient. MS lesions are generally categorized according to the stage of the demyelinating activity and the presence of immune cells (Frohman et al., 2006); active demyelinating lesions are characterized by the presence of myelin-laden macrophages in the lesion or at the edge; smoldering lesions contain rare myelin-phagocytosing macrophages at the rim of the lesion whereas chronic inactive lesions display no evidence for ongoing demyelination although single T cells may be present in perivascular regions (Frohman et al., 2006). Recently, several efforts have been done to understand the causes for remyelination failure. It has been postulated that in human active lesions (where remyelination is possible), the density of fibroblast growth factor-2 (FGF-2) expressing macrophages and FGF receptor 1 (FGFR1)-OPCs increase, whereas the extracellular matrix (ECM) associated glycoprotein anosmin-1 is absent. In contrast, in chronic-lesions (where remyelination is mainly restricted to the periplaque), FGF-2 expression is limited to the macrophages/microglia in the periplaque, and expression of anosmin-1 is widespread throughout the core of the lesion and may be preventing remyelination (Clemente et al., 2011). Multiple causes seem to contribute to the decline in remyelination, including the failure of OPCs to differentiate and to loop around the nude neuronal axons (Franklin et al., 2002). Regenerative approaches for demyelinating diseases have been based on the stimulation of the endogenous self-repair mechanisms, or on the transplantation of myelinogenic cells, as for instance derived from neural stem cells (NSCs; Grade et al., 2013).

NSCs from the SVZ can be highly expanded in culture and give rise to neurons, astrocytes and oligodendrocytes. Importantly, SVZ-derived oligodendrocytes undergo all the oligodendrocyte cell lineage stages from the early bipolar OPC to the mature myelinating oligodendrocyte, recapitulating the developmental process (Levison and Goldman, 1993; Menn et al., 2006). Furthermore, these cells are able to remyelinate vulnerable axons when transplanted in animal models of demyelinating injuries (Keirstead et al., 1999; Smith and Blakemore, 2000; Akiyama et al., 2001; Pluchino et al., 2003; Cayre et al., 2006). However, under normal conditions, oligodendrocytes represent a small minority of the SVZ progeny both *in vitro* and *in vivo* (Menn et al., 2006). Excitingly, the last decade has been considerably rich in developing tools to guide or force cells to acquire a desired fate. Indeed, pharmacological or genetic tools may be used to direct NSCs to the oligodendrocyte cell lineage, thus increasing the proportion of oligodendrocytes in the expanded cultures, prior to transplantation. On the other hand, the weak endogenous remyelination at chronic stages of the disease may be assisted by fostering oligodendrocyte differentiation since this constitutes a limiting step for effective remyelination. Thus, much interest has been gathered on finding molecular cues for oligodendrocyte lineage specification and development.

## MODULATION OF OLIGODENDROGENESIS

Oligodendrogenesis is regulated by intrinsic and environmental factors. A refined knowledge of these factors is necessary in order to be able to manipulate oligodendrogenesis for brain repair purposes in demyelinating diseases.

Importantly, the generation of induced OPCs (iOPCs) by direct lineage conversion was recently demonstrated. In fact mouse and rat fibroblasts can be reprogrammed by forced expression of oligodendrogenic transcription factors into iOPCs with morphologies and gene expression signatures resembling primary OPCs (Najm et al., 2013; Yang et al., 2013).

At the levels of gene expression, it has been shown that oligodendrogenesis is modulated by the histone deacetylase sirtuin 1 (SIRT1). Histone acetylation is mainly associated with active gene transcription. SIRT1 is a member of the sirtuin family and is expressed in adult mice SVZ and dentate gyrus (DG) in Sox2+ stem cells, proliferating cells and oligodendrocytes but not in mature astrocytes. Loss of SIRT1 deacetylase activity by conditional ablation of SIRT1 in nestin+ stem cells results in increased number of oligodendrocyte transcription factor 2 (Olig2)+ OPCs generated in the SVZ and migrating toward the septum and the striatum. In fact SIRT1 limits the expression of platelet-derived growth factor receptor alpha (PDGFRα) in nestin+ stem cells, involved in proliferation and differentiation of OPCs. Using chromatin immunoprecipitation, the authors showed that loss of SIRT1 increases histone 3 lysine 9 (H3K9) acetylation in the PDGFRα promoter, therefore allowing its transcription (Rafalski et al., 2013).

Expression of specific transcription factors is required to commit cells to the oligodendrocyte lineage. As an example, the basic-helix-loop-helix transcription factors Olig2 and Mash1 are key players in determining OPC specification. During postnatal oligodendrogenesis, OPCs double-labeled for Mash1 and Olig2, are found in the SVZ and corpus callosum. As maturation proceeds, OPCs express PDGFRα and down-regulate Mash1. Ablation of Mash1 during perinatal oligodendrogenesis in mice decreases the number of mature oligodendrocytes, in the dorsal telencephalon and corpus callosum. In fact, Mash1 regulates the division of PDGFRα+ OPCs inducing the asymmetric division of OPCs into one OPC and one oligodendrocyte. In the absence of Mash1, OPCs divide symmetrically into 2 OPCs decreasing the generation of future myelinating oligodendrocytes (Nakatani et al., 2013). During the perinatal period, conditional forced expression of Olig2 in nestin+ cells of the SVZ increases the number of OPCs in the SVZ and of mature and myelinating oligodendrocytes in the corpus callosum (Maire et al., 2010). These data demonstrate that Olig2 expression is crucial for the specification of SVZ nestin+ stem cells and OPCs differentiation. Also, a recent study highlights the importance of Musashi1 function in oligodendrogenesis. In fact, Musashi1 mRNA binding protein is expressed in the SVZ stem cells and is required for the maintenance of the stem cell fate notably by inhibiting the translation of the Notch inhibitor Numb. Moreover, retroviral-mediated transduction of Musashi1 in lumbar dorsal funiculus of mouse pups decreases the proportion of mature oligodendrocytes (Dobson et al., 2008).

Many soluble factors have been shown to direct oligodendrogenesis from OPCs and NSCs. For instance, the intracerebroventricular infusion of epidermal growth factor (EGF) increases OPCs production in the SVZ, which migrate and differentiate into myelinating oligodendrocytes in the corpus callosum, striatum and fimbria-fornix (reviewed in Gonzalez-Perez and Alvarez-Buylla, 2011). The pigment epithelium-derived factor (PEDF) is another pro-oligodendrogenic factor. PEDF is secreted by endothelial cells and ependymal cells in the SVZ and acts on stem cells to promote self-renewal (Ramirez-Castillejo et al., 2006). Intracerebral injection of PEDF increases the number of OPCs in the SVZ without affecting proliferation, but rather suggesting a role for PEDF in promoting oligodendroglial specification. In fact, PEDF infusion induces the survival and maturation of local OPCs in the corpus callosum into myelinating oligodendrocytes. *In vitro*, PEDF increases the commitment of glial fibrillary acidic protein (GFAP)+ stem cells toward the oligodendrocyte lineage by inducing the expression of transcription factors related to oligodendrogenesis such as Sox10, Olig1 and Olig2 (Sohn et al., 2012). Similarly, FGF-2 signaling is crucial during the onset of perinatal oligodendrogenesis in the SVZ. Intraventricular injection of FGF-2 increases proliferation and OPCs production in the SVZ (Azim et al., 2012). Surprisingly conditional inactivation of FGFR-2 signaling in oligodendrocytes does not affect normal brain development in mice (Kaga et al., 2006). Nevertheless, FGFR1 and FGFR2 signaling play a role in the specification of OPCs from the embryonic ventral forebrain and regulates myelin sheath (Furusho et al., 2011, 2012). Also, it has been shown that infusion of PDGF-AA into the ventricle induces the hyper-proliferation of NSCs, the so-called type B cells, expanding and producing hyperplasias of Olig2+ OPCs. Type B cells, but not actively proliferative progenitors (type C cells), express the receptor PDGFRα and therefore are the cells that originate these glioma-like structures (Jackson et al., 2006). Intracerebroventricular injection of the morphogen sonic hedgehog in adult mice brain increases cell proliferation in the SVZ, septum, striatum and cerebral cortex, and maturation of oligodendrocytes in the corpus callosum and cortex (Loulier et al., 2006). The neuroprotective factor thymosin β4 (Tβ4) present in neurons and microglia induces differentiation of SVZ into myelinating oligodendrocytes expressing myelin basic protein (MBP) and 2′,3′-cyclic-nucleotide 3′-phosphodiesterase (CNPase). Tβ4 inhibits both c-Jun expression and phosphorylation in SVZ cultures and therefore promotes oligodendrogenesis as phosphorylated c-Jun binds to myelin genes promoters such as MBP and CNPase to inhibit their expression (Santra et al., 2012). The thyroid hormone T3 is also a potent inducer of oligodendrocytic differentiation from SVZ cells (Whittemore et al., 1999; Grade et al., 2010).

The ECM also regulates oligodendrogenesis. During the perinatal wave of oligodendrogenesis, OPCs localize close to laminin-rich basal lamina of vessels in the SVZ. Genetic ablation of laminin alpha 2 subunit increases cell death of OPCs in the SVZ and in the corpus callosum during perinatal oligodendrogenesis. Later, by 3 weeks of age, an accumulation of OPCs but fewer mature oligodendrocytes is found and the myelin sheath is thinner. Therefore laminin is necessary for OPCs survival and maturation of OPCs into myelinating oligodendrocytes (Relucio et al., 2012). In fact, oligodendrocytes express a limited repertoire of integrins including α6β1, αvβ1, αvβ3, and αvβ5 that are the receptors for ECM laminin (α6β1), fibronectin and vitronectin. Sequential expression of these integrins regulates the timing of proliferation, migration and differentiation during oligodendrogenesis. Oligodendrocyte O-2A precursor cells are proliferative and migratory bipolar oligodendrocytes expressing α6β1, αvβ1, and αvβ3 integrins. Upon specific blocking of αv by Arg–Gly–Asp (RGD) peptides and anti-β1 antibodies, migration is blocked. During differentiation, oligodendrocyte precursors lose their ability to migrate which is correlated to a decrease of αvβ1 integrin expression (Milner and ffrench-Constant, 1994; Milner et al., 1996). At this step, αvβ3 integrin is transiently expressed and stimulates proliferation before being down-regulated. Indeed, enhanced proliferation is induced in CG-4 rat oligodendrocyte cell line engineered to express αvβ3 integrin and plated on vitronectin (and not on poly-D-lysine; Blaschuk et al., 2000). Then cells exit cell cycle and αvβ5 integrin is up-regulated for terminal differentiation and maturation (Milner and ffrench-Constant, 1994). In OPCs, integrins regulate biological outcomes of PDGF sensing. In fact, association of PDGFRα to αvβ3 integrin activates the Src kinase Lyn and triggers proliferation on fibronectin support. However, upon contact with axonal laminin to α6β1, the Src kinase Fyn is activated and potentiates prosurvival capacities of PDGFRα (Colognato et al., 2004). Moreover, α6β1 plays a crucial role in oligodendrocyte myelination. Numbers of wraps of myelin and amount of myelin protein MBP at axoglial contacts are dependent of the axon diameter. It has been shown that laminin substrate induces oligodendrocytes differentiation with increase in processes complexity and membrane expression of MBP as compared to oligodendrocytes plated on poly-D-lysine (Laursen et al., 2011). This effect is due to translation of MBP mRNA located in the myelinating nodes. The 3′UTR region of the MBP mRNA contains elements that repress its translation notably by binding the mRNA binding protein heterogeneous nuclear ribonucleoprotein-K (hnRNP-K). In the presence of active α6β1 integrin (through contact with axonal laminin), this inhibition is abolished. By immunoprecipitation, it was shown that hnRNP-K interacts with α6β1 integrin in mature oligodendrocytes cultured on poly-D-lysine and this interaction is decreased on laminin substrate. Laursen et al. (2011) propose that upon activation of integrins, phosphorylation of hnRNP-K induces the release of MBP mRNA for translation and myelination.

## MODULATION OF OLIGODENDROGENESIS BY ENDOCANNABINOIDS

Cannabinoids act on at least two types of receptors, type 1 and type 2 cannabinoid receptors (CB1R and CB2R), which are predominantly distributed in the CNS and immune system, respectively (Mackie, 2008). Although low levels of CB2R have also been detected in the CNS (Onaivi et al., 2006). In the brain, CBR are targeted by endocannabinoids such as anandamide (AEA) and 2-arachidonylglycerol (2-AG), which are molecules produced from membrane lipid precursors in response to cell activation, and therefore a reaction tightly controlled by neuronal activity (Galve-Roperh et al., 2008). Once generated, endocannabinoids act retrogradely mainly through presynaptic CB1R, blunting membrane depolarization and inhibiting neurotransmitter release (Galve-Roperh et al., 2008).

Interestingly, it was found that CB1R and CB2R are expressed *in vivo* in the SVZ and DG and in nestin+ cells from neural progenitor cultures (Jin et al., 2004; Aguado et al., 2005; Arevalo-Martin et al., 2007; Molina-Holgado et al., 2007). In fact, it was shown that CB1R and CB2R activation promotes NSC proliferation both in the hippocampus and SVZ (Jin et al., 2004; Aguado et al., 2005; Palazuelos et al., 2006; Molina-Holgado et al., 2007; Goncalves et al., 2008; Xapelli et al., 2013). Moreover, endocannabinoids have emerged as a potential target to modulate oligodendrogenesis, by modulating CB1R and CB2R. In fact, Arevalo-Martin et al. (2007) have found that postnatal treatment with a CB1R agonist increased the number of Olig2+ cells in the rat SVZ. Furthermore, recent studies suggest that endocannabinoids play a role in oligodendrocytic differentiation (Gomez et al., 2010; Solbrig et al., 2010; Gomez et al., 2011) and might modulate several neurodegenerative diseases (reviewed in Velayudhan et al., 2013). Very recently it was shown that CB1R activation promoted differentiation of OPCs and improved remyelination after stroke-induced demyelination (Sun et al., 2013). Despite the role of endocannabinoid receptors in several pathologies, selective agonists and antagonists produce severe adverse side effects. Indeed, activation of CB1 and/or CB2 receptor leads to increased psychoactivity, cardiovascular problems, obesity, diabetes, and inflammation (reviewed in Pacher and Kunos, 2013).

Hence it is of great importance to find alternative new drugs able to modulate cannabinoid receptors. Recent findings suggest that hemopressin (Hp), a nine residue-long peptide derived from the hemoglobin (Hg), modulates the cannabinoid receptors activity (Heimann et al., 2007; Gomes et al., 2010; Bomar and Galande, 2013). In fact, it was shown that Hp acts as a CB1R inverse agonist *in vivo* and inhibits appetite and induces antinociception (Heimann et al., 2007; Dodd et al., 2010) and importantly it was suggested that Hp exerts its effects by an alternative mode of action which might avoid adverse side effects (Dodd et al., 2013). Therefore, we investigated the role of Hp in SVZ oligodendrogenesis and found that this peptide promotes oligodendrocytic differentiation and maturation.

## MATERIAL, METHODS AND RESULTS

All experiments were performed in accordance with the European Community guidelines for the care and use of laboratory animals (86/609/EEC; 2010/63/EU).

**FIGURE 1:**
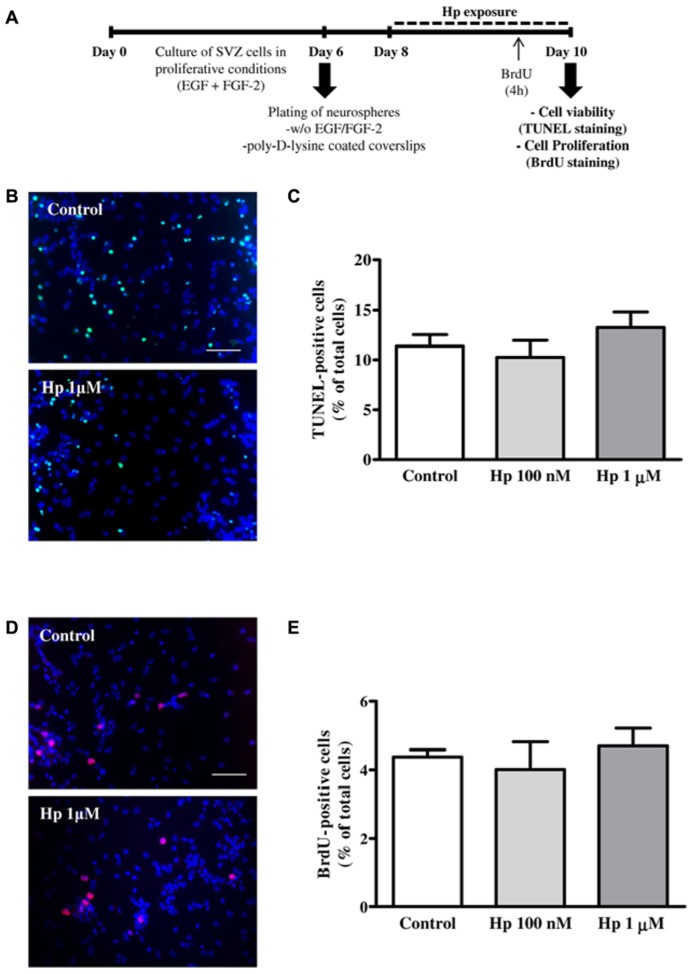
**Hp did not induce cell death nor cell proliferation. (A)** Experimental protocol. **(B)** Representative fluorescence digital images of SVZ cell cultures treated for 48 h in the absence (control) or presence of Hp (1 μM) and stained using the TUNEL (Terminal deoxynucleotidyl transferase dUTP nick end labeling) method to reveal apoptotic nuclei (in green) and Hoechst 33342 (blue nuclei). **(C)** Bar graph depicts the percentages of TUNEL-stained nuclei. **(D)** Representative fluorescence digital images of SVZ cell cultures in the absence (control) or presence of Hp (1 μM) and immunolabeled for BrdU (5-bromo-2′-deoxyuridine, red nuclei) and Hoechst 33342 (blue nuclei). **(E)** Bar graph depicts the percentages of BrdU-immunostained nuclei. Data are expressed as a mean ± SEM of 2–3 independent experiments. Scale bar: 50 μm.

**FIGURE 2:**
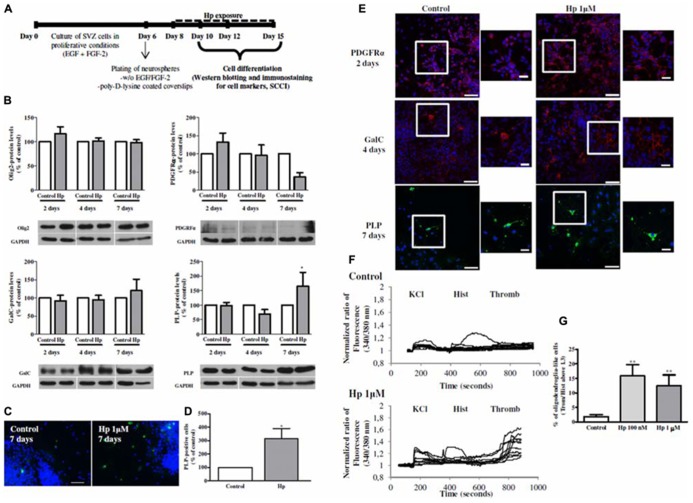
**Hp induced oligodendroglial differentiation in SVZ cultures. (A)** Experimental protocol. **(B)** Bar graphs of oligodendrocyte transcription factor 2 (Olig2), Platelet-derived growth factor receptor alpha (PDGFRα), Galactosylceramidase (GalC) and Proteolipid Protein (PLP) protein levels of control and Hp 1 μM treated cultures for 2, 4, and 7 days. Data are expressed as mean ± SEM of 4–7 independent experiments. *P < 0.05; using Bonferroni’s multiple comparison test for comparison with the respective control culture. **(C)** Representative fluorescence digital images of PLP+ oligodendrocytes (in green) and Hoechst 33342 staining (blue nuclei) in SVZ cultures. Scale bar: 50 μm. **(D)** Bar graph depicts the number of PLP+ cells, expressed as the total number of cells counted per culture, in control and Hp (1 μM) treated cultures for 7 days. Data are expressed as mean ± SEM of 4 independent experiments. *P < 0.05 using using unpaired student’s *t*-test for comparison with control culture. **(E)** Representative confocal digital images of PDGFRα, GalC and PLP in control cultures and in cultures treated with Hp 1 μM for 2, 4, and 7 days of differentiation, respectively. PDGFRα (in red); GalC (in red), PLP (in green), and Hoechst 33342 (used to visualize cell nuclei, in blue). Scale bars = 50 μm. Squares are plans of higher magnifications. Scale bars = 20 μm. **(F)**: Representative single cell calcium imaging (SCCI) profiles of response of 8–10 cells in a control culture (no drug exposure) and, in a culture treated with Hp (1 μM) for 7 days. **(G)** Bar graph depicts the percentages of oligodendroglial-like responding cells in SVZ control cultures and in cultures exposed to Hp (100 nm and 1 μM). Data are expressed as mean ± SEM of 4–7 independent experiments. **P < 0.01; using Dunnett’s multiple comparison test for comparison with SVZ control cultures.

We have used SVZ neurospheres prepared from early postnatal (P1-3) C57BL/6 mice in serum-free medium (SFM) supplemented with 10 ng/ml EGF and 5 ng/ml FGF-2 (both from Invitrogen, Carlsbad, CA, USA), as described previously (Xapelli et al., 2013). SVZ neurospheres were seeded for 48 h onto glass coverslips coated with 0.1 mg/ml poly-D-lysine in SFM medium devoid of growth factors. To evaluate cell death/proliferation or oligodendrocytic differentiation, the neurospheres were allowed to develop for further 2, 4, and 7 days at 37°C in the absence or presence of Hp (100 nM or 1 μM; Proteimax, São Paulo, SP, Brazil; **Figures 1A** and **2A)**.

First we investigated whether Hp modified cell apoptosis, which was evaluated by the terminal deoxynucleotidyl transferase dUTP nick-end labeling (TUNEL) assay. SVZ plated neurospheres treated with Hp (100 nM or 1 μM) for 48 h (**Figure 1A**) were fixed in 4% paraformaldehyde (PFA) and reacted with the terminal deoxynucleotidyl transferase according to manufacturer’s instructions (Roche, Basel, Switzerland). Apoptotic nuclei were labeled with fluorescein followed by nuclei counterstaining with Hoechst 33342 (all from Invitrogen). Finally, the preparations were mounted using Dakocytomation fluorescent medium (Dakocytomation, Carpinteria, CA, USA). We observed no significant differences in the percentage of TUNEL+ nuclei between control and Hp-treated cultures, indicating that Hp was not toxic to the cells (Control: 11.38 ± 1.15%, Hp 100 nM: 10.26 ± 1.70%, Hp 1 μM: 13.25 ± 1.56%; *n* = 3; **Figures 1B,C**).

To investigate the effect of Hp on cell proliferation, SVZ cells were exposed to 10 μM 5-bromo-2’-deoxyuridine (BrdU; Sigma-Aldrich), for the last 4 h of each Hp (100 nM or 1 μM) treatment (48 h). Then, SVZ cells were fixed in PFA and BrdU was immunolabeled with the anti-BrdU mouse monoclonal antibody (Clone MoBU-1) Alexa Fluor 594 (Invitrogen) as described previously (Xapelli et al., 2013; **Figure 1A**). We have found that Hp did not induce proliferation since the percentage of BrdU+ cells in Hp treated cultures were similar to control conditions (Control: 4.38 ± 0.21%, Hp 100 nM: 4.01 ± 0.81%, Hp 1 μM: 4.70 ± 0.53%; *n* = 2; **Figures 1D,E**).

Hp effect on oligodendroglial differentiation was investigated by incubating SVZ neurospheres for 2, 4, or 7 days with Hp (1 μM; **Figure 2A**) and western blotting analysis of oligodendrocyte markers at different stages of differentiation was performed: Olig2 (present during all oligodendrocyte development), PDGFRα (marker of OPC or pre-oligodendrocyte), Galactosylceramidase (GalC, pre-myelinating oligodendrocyte) and proteolipid protein (PLP, myelinating oligodendrocyte). Moreover, an immunocytochemistry for PDGFRα, GalC and PLP was performed following 2, 4, and 7 days of Hp treatment, respectively with the primary antibodies listed in **Table 1** and using a protocol described previously (Xapelli et al., 2013).

**Table 1:** Primary antibodies used for immunocytochemistry.

Antigen	Company	Host	Dilution
**PDGFRα**	BD Biosciences, San Diego, CA, USA	Rat	1:500
**GalC**	Millipore	Mouse	1:500
**PLP**	AbD Serotec, Kidlington, UK	Mouse	1:1000

We have observed that although most of protein levels did not change when comparing with the respective control at the different time-points (**Figure 2B**), the morphology was very different in the cultures that were treated with Hp 1 μM (**Figure 2E**). In fact, PDGFRα and GalC immunoreactivity was increased following Hp treatment for 2 and 4 days respectively (**Figure 2E**). Interestingly, after 7 days of Hp incubation PLP-protein levels increased (to 165.3 ± 47.77% when compared with 100% Control 7 days, *n* = 4, *P* < 0.05; **Figure 2B**). Moreover, we observed that oligodendrocyte maturation was promoted upon Hp treatment for 7 days since the number of PLP+ cells increased when compared with control cultures (Control: 100 ± 0.00%, Hp 1 μM: 314.40 ± 76.06%; *n* = 4, *P* < 0.05; **Figures 2C–E**).

Functional oligodendroglial differentiation was evaluated by single cell calcium imaging (SCCI) to analyze the intracellular variations of calcium-free levels ([Ca^2^^+^]_i_) in single cells following stimulation with 50 mM KCl, 100 μM histamine (Sigma-Aldrich) and 0.1 U/ml thrombin (Sigma-Aldrich) as previously described (Grade et al., 2010). Briefly, SVZ cultures were loaded with Fura-2/AM, then were continuously perfused with Krebs solution (132 mM NaCl, 4 mM KCl, 1.4 mM MgCl_2_, 1 mM CaCl_2_, 6 mM glucose, 10 mM HEPES, pH 7.4) and stimulated with KCl, histamine and thrombin. [Ca^2^^+^]_i_ variations were evaluated by quantifying the ratio of the fluorescence emitted at 510 nm following excitation at 340 and 380 nm. KCl, histamine, and thrombin peaks given by the normalized ratios of fluorescence at 340/380 nm, were used to calculate the ratios of the responses to thrombin/histamine, as it was previously shown that ratios above 1.3 are consistent with oligodendroglial differentiation in SVZ cultures (Grade et al., 2010). Indeed it was shown that cells that increased their calcium levels specifically following thrombin stimulation are immunoreactive for O4 and PLP; and oligodendrocytes progenitors expressing the proteoglycan NG2 are responsive to thrombin (lesser than mature oligodendrocytes) but also to histamine consistent with a transition state between immature precursor and mature oligodendrocytes (Grade et al., 2010). In fact, Hp treatment increased the percentage of oligodendrocyte-like cells in SVZ cell cultures (Control: 1.83 ± 0.73%, Hp 100 nM: 15.84 ± 3.85%, Hp 1 μM: 12.44 ± 3.76%; *n* = 4, *P* < 0.01; **Figures 2F,G**). Moreover, using single cell imaging, we quantified the number of cells displaying a KCl/Histamine ratio of response inferior to 0.8 (consistent with mature neurons), and a ratio between 0.9 and 1 (consistent with mature GFAP+ astrocytes; Agasse et al., 2008). We found that Hp at 1 μM for 7 days increase neuronal differentiation (Control: 11.65 ± 1.58%; Hp 100 nM: 22.68 ± 7.99%; Hp 1 μM: 22.52 ± 5.68%; *n* = 6, *P* < 0.05), without altering the number of GFAP-positive astrocytes (Control: 40.01 ± 3.39%; Hp 100 nM: 34.40 ± 4.64 %; Hp 1 μM: 33.73 ± 3.32; *n* = 6; data not shown).

## FUTURE DIRECTIONS

Due to the adverse side effects of synthetic cannabinoid agonists and antagonists (reviewed in Pacher and Kunos, 2013) it is very important to find other selective modulators that maintain therapeutic properties without producing side effects. Therefore, Hp, as a peptide modulator of the cannabinoid receptor, may be a potential therapeutic agent. In fact, it was shown that Hp inhibits appetite and induces antinociception without causing motor abnormalities or sedative effect (Heimann et al., 2007; Dodd et al., 2010, 2013).

Here we have observed that Hp treatment did not affect cell death and proliferation, but promoted oligodendrocytic differentiation and maturation of SVZ stem/progenitor cells. In light of these evidences, Hp and its derivates might be useful in future strategies to treat or prevent demyelinating disorders. Therefore, following steps urge at studying the effect of Hp in animal models of demyelinating diseases, from oligodendrocytic differentiation *in vivo* to amelioration of the disease symptoms.

## AUTHOR CONTRIBUTIONS

Sara Xapelli: Conception and design; Administrative support; Provision of study material; Collection and/or assembly of data; Data analysis and interpretation; Manuscript writing; Final approval of manuscript. Fabienne Agasse: Conception and design; Financial support; Collection and/or assembly of data; Data analysis and interpretation, Manuscript writing; Final approval of manuscript. Sofia Grade: Manuscript writing. Liliana Bernardino: Financial support; Administrative support; Manuscript writing. Filipa F. Ribeiro and Clarissa S. Schitine: Provision of study material; Collection and/or assembly of data. Ricardo A. De Melo Reis: Conception and design; Provision of study material; Collection and/or assembly of data. Andrea S. Heimann and Emer S. Ferro: Conception and design; critical reading of manuscript. Ana M. Sebastião: Financial support; Final approval of manuscript. João O. Malva: Conception and design; Financial support; Data analysis and interpretation; Critical reading of manuscript; Final approval of manuscript.

## Conflict of Interest Statement

The authors declare that the research was conducted in the absence of any commercial or financial relationships that could be construed as a potential conflict of interest.
